# Poor treatment outcomes and associated factors among hospitalized patients with stroke at Hiwot Fana Comprehensive Specialized Hospital, eastern Ethiopia

**DOI:** 10.3389/fstro.2023.1304664

**Published:** 2023-12-08

**Authors:** Zerihun Abera Ayele, Sisay Molla, Aliyi Ahmed, Teshager Worku, Addisu Seneshaw Bezabih, Biniyam Tedla Mamo

**Affiliations:** ^1^Department of Internal Medicine, College of Health and Medical Science, Jigjiga University, Jigjiga, Ethiopia; ^2^Department of Internal Medicine, College of Health and Medical Science, Haramaya University, Harar, Ethiopia; ^3^School of Nursing and Midwifery, College of Health and Medical Science, Haramaya University, Harar, Ethiopia; ^4^Arada Giorgis Specialized Health Center, Addis Ababa, Ethiopia

**Keywords:** adult stroke, cerebrovascular accident, Ethiopia, stroke, treatment outcome

## Abstract

**Background:**

Stroke is a significant health problem in both industrialized and developing nations. It is the world's second-leading cause of death worldwide. Stroke incidence, prevalence, and death rates have grown internationally, with low- and middle-income nations suffering the greatest proportion of the burden. Stroke is a leading cause of long-term physical impairment, affecting a person's quality of life, societal engagement, independence, emotions, and productivity.

**Objective:**

To determine the magnitude and factors associated with poor treatment outcomes in hospitalized adult patients with stroke.

**Methods:**

A hospital-based cross-sectional study was conducted from January 2019 to June 2021 in stroke patients admitted to the Hiwot Fana Comprehensive Specialized Hospital. This study included 290 patient charts. Data were collected by reviewing the medical charts using a well-developed data abstraction form. Data were entered into Epi-Data version 3.2 and exported to SPSS version 25.0. Descriptive statistics were used to describe study variables. Additionally, bivariable and multivariable logistic regression analyses were used to identify factors associated with poor stroke treatment outcomes. All statistical tests were set at 5% of significant threshold.

**Results:**

Among 290 enrolled patients, 172 (59.3%) had poor stroke treatment outcomes. The mean age of the patients was 54.7(*SD*: ±16.1) years, and more than half 182 (62.8%) of the participants were males. The overall average length of hospital stays for stroke patients was 8 ± 3.3 days. Age of 45–64 years (adjusted odds ratio [AOR]: 2.17, 95% CI [1.06, 4.41]), aspiration pneumonia (AOR: 2.13, 95% CI [1.06, 4.26]), systolic blood pressure ≥ 140 mm Hg/dl (AOR: 2.35, 95% CI [1.24, 4.47]), Glasgow Coma Scale score of <8 (AOR: 7.26, 95% CI [3.82, 13.8]), and serum creatinine level of ≥1 mg/dl (AOR: 2.73, 95% CI [1.46, 5.10]) were significantly associated with poor treatment outcome in adult stroke patients.

**Conclusion:**

Six out of ten stroke patients had poor treatment outcomes. Age between 45 and 65 years, uncontrolled hypertension, aspiration pneumonia, low Glasgow Coma Scale score at admission, and renal injury were identified as significantly associated with poor treatment outcomes in stroke patients.

## Introduction

According to the WHO stroke definition, stroke is confirmed in the presence of “rapidly developed clinical signs of focal (or global) disturbance of cerebral function, lasting more than 24 h or leading to death, with no apparent cause other than of vascular origin” (Bindslev et al., 2023). Stroke is a primary cause of significant, long-term physical disability, with long-term detrimental effects on quality of life, social life, independence, emotions, and productivity. In the absence of a strong global public health response, the worldwide burden of stroke is expected to exceed 23 million new cases and 7.8 million fatalities per year by the end of 2030 (Hilari, 2011; van Eeden et al., 2012; Satink et al., 2013; De Wit et al., 2017; Avan et al., 2019; Mairami et al., 2020).

Stroke epidemiology's worldwide burden and clinical consequences are rapidly evolving. Stroke prevalence in low- and middle-income countries (LMICs) has more than doubled over the previous four decades, whereas stroke incidence in wealthy nations has fallen by 42%. Furthermore, LMICs have the greatest stroke mortality rate, accounting for 5 million fatalities per year. The majority of stroke deaths occur in LMICs, indicating that the developing world suffers the greatest burden of stroke mortality, morbidity, and disability (Bindslev et al., 2023). Research has also revealed that, in LMICs, there is low use of evidence-based practice for acute stroke care (Alene et al., 2020). In 2015, the age-standardized annual stroke incidence rate in Africa was 316 per 100,000 persons, with an age-standardized prevalence of 981/100,000 (Owolabi et al., 2015).

Stroke accounts for 4.71% of all fatalities, according to WHO data published in 2014 (Johnson et al., 2016). A large number of studies on the epidemiology of stroke in Ethiopia have indicated important differences in the clinical and demographic characteristics of patients compared to those in developed nations. A higher percentage of hemorrhagic stroke than ischemic stroke has been reported in Ethiopian hospitals. In Ethiopia, the burden and outcomes of ischemic and hemorrhagic stroke vary by area and time period (Alene et al., 2020; Baye et al., 2020; Abate et al., 2021). The majority of deaths in Ethiopia occur shortly after admission due to the severe neurologic and medical consequences of stroke. In-hospital mortality is increased due to late presentation, poor care, and insufficient rehabilitation services, and the vast majority of patients leave the hospital with significant physical disabilities (Deresse and Shaweno, 2015; Shenkutie Greffie, 2015; Alene et al., 2020; Baye et al., 2020; Abate et al., 2021).

There is limited data on the burden of stroke, its risk factors, and treatment outcomes in our country, especially in eastern Ethiopia. Therefore, this study assessed the treatment outcomes and factors associated with stroke that would be essential to strengthen and revitalize existing health care policies and strategies to reduce the burden of stroke in the region and identify areas of improvement in the care of stroke patients.

## Methods and materials

### Study design and study area

This institutional-based cross-sectional study was conducted from January 2019 to June 2021. This study was carried out at Hiwot Fana Specialized University Hospital (HFSUH), which is located in Harar, the capital city of Harari regional state, 526 kilometers from Ethiopia's capital, Addis Ababa. HFSUH is one of the region's two public hospitals. The hospital serves as a referral hospital for the Harari regional state and Oromia's East Hararghe Zone. It also serves more than 5.2 million people.

### Eligibility criteria

Stroke patients hospitalized in the HFSUH neurology ward between January 1, 2019, and June 30, 2021, and diagnosed clinically and confirmed by brain imaging as stroke were included in the study. Patients younger than age 18, those with inadequate medical records, and those with a transient ischemic attack were all excluded from the research.

### Sample size determination

To select eligible patients for the research study, a survey of all medical records of stroke patients hospitalized between January 2019 and June 2021 was done. As a result, 290 medical records from stroke patients were included in the study. A logbook was used to compile a list of all card numbers of stroke patients admitted to HFSUH during the research period. Then, we used a convenience sampling approach to get the total number of research participants, which was 290.

### Statistical analysis

Collected data were manually checked for completeness and consistency. Data were entered into Epi-Data version 3.2 and analyzed using SPSS version 25.0. Descriptive statistics, including frequency, percentage, mean, and standard deviation, were used to describe the variables. Bivariable logistic regression was used to assess the association between each independent and dependent variable, and multivariable logistic regression analyses were used to identify factors significantly associated with poor stroke treatment outcomes. Adjusted odds ratios (AOR) and 95% confidence intervals were used to measure the strength of association. A multicollinearity test was performed to check the correlation between independent variables using the variance inflation factor (VIF). The model fitness, the Hosmer–Lemeshow test, was used to determine model fitness. Finally, a *p*-value of <0.05 was used to declare the presence of a significant association in multivariable logistic regression.

### Operational definitions

Stroke: Stroke is defined by the WHO as “rapidly developing clinical signs that result in focal or global disturbance of cerebral function, lasting 24 hours or longer, or leading to death with no known cause other than vascular origin.”

Poor treatment outcomes: The patient is discharged with complications, referred to a higher health facility, or left against medical advice or death (Kefale et al., 2020).

Good treatment outcomes: When the patient was discharged, complaints and physical findings were reduced compared with the time of admission (Kefale et al., 2020).

Glasgow Coma Scale: The Glasgow Coma Scale (GCS) is used to assess the state of consciousness in patients with stroke. A good GCS assessment is described as mild brain injury/alert (GCS score of 13–15) or severe brain injury/drowsy (GCS score of 9–12). If the patient has serious brain injury/unconsciousness, a GCS score ≤8 is considered poor (Zewudie et al., 2020). For this study purpose, we classified GCS scores in two groups: <8 and >8.

## Result

### Sociodemographic characteristics

The average age of the patients was 54.7(*SD*: ±16.1) years, and 38.3% of the participants were in the 45–64-years-old age group. More than half, 182 (62.8%), were males, and the majority, 206 (71.0%), were rural residents (Table 1).

**Table 1:** Sociodemographic characteristics of adult stroke patients admitted to Hiwot Fana Specialized University Hospital.

**Variables**		**Frequency**	**Percentage**
Age (in years)	≤ 44	79	27.2
	45–64	111	38.3
	≥65	100	34.5
Sex	Male	182	62.8
	Female	108	37.2
Residence area	Rural	206	71.0
	Urban	84	29.0
Time of admission or year of illness	2021	64	22.0
	2020	128	44.1
	2019	98	33.9

### Clinical characteristics of study participants

One hundred seventy (58.6%) adult stroke patients had an ischemic stroke. Of the 170 patients with ischemic stroke, 30 (17.6%) had a documented cardioembolic stroke. A majority, 227 (78.3%), of the study participants visited the health facility within 48 h of the onset of the first symptom, and the median time to visit the facility from the onset of the first symptom was 24 h. The overall average length of hospital stay (LOS) is 8 ± 3.3 days. The mean LOS for a patient with a poor treatment outcome was 6 ± 2.5 days. A total of 273 (94.1%) patients had focal neurologic deficits, and 142 (49.0%) had comorbidities at admission. Regarding the baseline comorbidities of the patients, nearly one-third, 103 (35.5%), had hypertension. Aspirin (56.6%), statins (59.3%), antihypertensive medications (57.9%), and antibiotics (42.8%) were the most commonly given medical treatments. Among previously diagnosed hypertensive patients, only 36 (34.9%) were taking antihypertensive medications (Table 2).

**Table 2:** Clinical characteristics of adult stroke patients admitted in Hiwot Fana Comprehensive Specialized Hospital, eastern Ethiopia (*n* = 290).

**Variables**		**Frequency (*n*)**	**Percentage**
Type of Stroke	Hemorrhagic stroke	120	41.4
	Ischemic stroke	170	58.6
Time from first symptom to hospital visit (hours)	≤ 48 h	227	78.3
	>48 h	63	21.7
Length of hospital stay	< 7 days	114	39.6
	>7 days	176	60.4
Presence of focal neurologic deficit at admission	Yes	273	94.1
	No	17	5.9
Presence of documented aspiration pneumonia	Yes	110	37.8
	No	180	62.1
History of previous stroke	Yes	28	9.7
	No	262	90.3
Comorbidity	Yes	142	49.0
	No	148	51.0
Being on treatment for a diagnosed hypertension	Yes	36	12.4
	No	254	87.6
SBP at admission (mm Hg)	≥140	127	43.8
	120–139	20	13.8
	< 120	123	42.3
DBP at admission	≥90	56	19.3
	61–89	170	58.6
	≤ 60	64	22.1
GCS score at presentation	>8	130	44.8
	≤ 8	160	55.2
Treatments are given after admission	Aspirin	164	56.6
	Statin	172	59.3
	Antihypertensive	168	57.9
	Mannitol	74	25.5
	Antibiotic	124	42.8
	Antiepileptics	62	21.4
	Any other drugs^*^	262	90.3

SBP, systolic blood pressure; DBP, diastolic blood pressure; GCS, Glasgow Coma Scale.

* Other drugs include protein pump inhibitors, anti-pain medications, and sedatives.

One hundred four (35.9%) adult stroke patients had serum creatinine (recent) >1 milligram per deciliter. One hundred twenty-two (42.1%) and 62 (57.2%) adult stroke patients had abnormal electro cardiogram (ECG) and echocardiography findings, respectively, and only 15 (5.2%) study participants had abnormal carotid Doppler ultrasound findings.

### Treatment outcome of stroke

A total of 172 (59.3 %) patients had poor stroke treatment outcomes. Of the 172 patients with poor outcomes, 50 (29.1%) died, 23 (13.3%) were discharged with the same condition as the time of admission, 92 (53.5%) were left against medical advice, and the remaining 7 (4.1%) were referred to another hospital.

### Factors associated with treatment outcome of stroke

Bivariable logistic regression was used to assess the association between independent variables and poor treatment outcomes among adult patients with stroke, and the variables age, sex, residence, focal neurologic deficit, comorbidity at admission, aspiration pneumonia, GCS score at presentation, systolic blood pressure (SBP) at admission, serum creatinine level, abnormal ECG were associated with a poor treatment outcome at a *p* < 0.25, which was selected for the multivariable regression (Table 3).

**Table 3:** Multivariable logistic regression of factors associated with poor treatment outcome.

**A variables**		**Treatment outcome of stroke**	**COR [95% CI]**	**AOR [95% CI]**	***p*-value**
		**Poor outcome**, ***n*** **(%)**			
Age (in years)	≤ 44	42 (53.2)	Ref	Ref	
	45–64	68 (61.3)	1.39 [0.78, 2.50]	2.17 [1.06, 4.41]	0.033
	≥65	62 (62.0)	1.44 [0.79, 2.62]	0.77 [0.35, 1.70]	0.513
Sex	Male	115 (63.2)	1.54 [0.95, 2.49]	1.41 [0.77, 2.59]	0.268
	Female	57 (52.6)	Ref	Ref	
Residence area	Urban	57 (67.9)	1.67 [0.98, 2.85]	**2.63 [1.30, 5.31]** ^*^	0.007
	Rural	115 (55.8)	Ref	Ref	
Focal neurologic deficit at admission	Yes	165 (60.4)	2.18 [0.81, 5.91]	1.17 [0.34, 4.08]	0.830
	No	7 (41.2)	Ref	Ref	
Aspiration pneumonia	Yes	85 (77.3)	3.63 [2.13, 6.20]	**2.13 [1.06, 4.26]** ^*^	0.001
	No	87 (48.3)	Ref	Ref	
Comorbidity at admission	Yes	110 (63.6)	1.55 [0.96, 2.50]	1.12 [0.57, 2.21]	0.742
	No	62 (53.0)	Ref	Ref	
SBP at admission (mm Hg)	≥140	89 (70.1)	1.77 [1.05, 2.99]	**2.35 [1.24, 4.47]** ^*^	0.009
	120–139	13 (32.5)	0.37 [0.17, 0.77]	**0.29 [0.12, 0.70]** ^*^	0.006
	< 120	70 (56.5)	Ref	Ref	
GCS score at presentation	≤ 8	125 (78.1)	6.31 [3.76,10.59]	**7.26 [3.82, 13.82]** ^*^	0.001
	>8	47 (36.2)	Ref	Ref	
Serum creatinine (recent) in mg/dl	≥1	72 (69.2)	1.94 [1.16, 3.21]	**2.73 [1.46, 5.10]** ^*^	0.002
	< 1	100 (53.8)	Ref	Ref	

HS, hemorrhagic stroke; IS, ischemic stroke; COR, crude odds ratio; AOR, adjusted odds ratio; SBP, systolic blood pressure; DBP, diastolic blood pressure; GCS, Glasgow Coma Scale; Ref, reference. ^*^Statistical significance at *p* < 0.0.

In the multivariate analysis, only six variables were identified as predictors of poor treatment outcomes. The 45–64-years-old age group (AOR: 2.17; 95% CI [1.06, 4.41]), urban residence (AOR: 2.63; 95% CI [1.30, 5.31]), documented aspiration pneumonia (AOR: 2.13; 95% CI [1.06, 4.26]), SBP ≥ 140 mm Hg (AOR: 0.29; 95% CI [0.12, 0.70]), GCS score ≤ 8 (AOR: 7.26; 95% CI [3.82, 13.8]), and serum creatinine ≥ 1 mg/dl (AOR: 2.73; 95% CI [1.46, 5.10]) were significantly associated with poor treatment outcomes in stroke patients (Table 3).

## Discussion

In this study, we analyzed the treatment outcomes and associated variables among adult stroke patients admitted to HFSUH in eastern Ethiopia. Accordingly, poor treatment outcomes among adult stroke patients were prevalent in the study area. Age 45–64 years, urban residence, aspiration pneumonia, high systolic blood pressure upon admission, and high serum creatinine were shown to be independent indicators of poor treatment outcomes among adult stroke patients.

The study showed that the mean age of stroke presentation is 54.7 years, which is lower than cases admitted in many European countries, where the average age at admission is 73 years. To the contrary, a similar study done in Africa found that the mean age at which patients have a stroke was lower than in other parts of the world: 55 years in Zambia, 58 years in Gambia, and 60 years in Senegal (Atadzhanov et al., 2012). Within their fourth or fifth decade of life, many Africans experience a stroke, which can have a major impact on the person who suffers the stroke, their family, and society as a whole. This age distribution is especially significant since younger stroke victims often experience a higher loss of self-worth and socioeconomic productivity than older victims (Akinyemi et al., 2021).

The epidemiology of ischemic stroke, the most common cerebrovascular accident subtype, was 58.6%. This result is aligned with that of studies from the Nekemte Referral Hospital (52.7%) (Fekadu et al., 2020). In our study, 59.3% of adult stroke patients have poor treatment outcomes in comparison to 37.8% in central Ethiopia, 48.1% Debre Markos referral hospital in northwestern Ethiopia, and 46.2% in Felege Hiwot comprehensive specialized hospital, respectively (Kefale et al., 2020; Mulugeta et al., 2020; Gadisa et al., 2021). However, in another study conducted at St. Paul Millennium Medical College, the rate of poor treatment outcomes was 75.5% (Tesfaye, 2017). These disparities can be explained by the large sample size used in our study compared to studies that reported a lower prevalence of poor treatment outcomes. The research showed that the prevalence of poor treatment outcomes among hemorrhagic stroke patients (60.8%) was roughly comparable to the prevalence of poor treatment outcomes among those with ischemic stroke (58.2%).

The most commonly prescribed medicines at HFSUH are statins (59.3%), antihypertensive therapy (57.9%), and aspirin (56.6%), The findings are consistent with research conducted in southwestern Ethiopia (Zewudie et al., 2020). However, these numbers are lower than that in a study conducted on the prescription pattern of drugs in stroke patients in India (Abbasi and Ali, 2012). Mannitol is the mainstay management of intra cranial pressure (ICP), particularly for hemorrhage stroke; 25.5% of patients received treatment during their hospital stay. This result is lower than the one obtained in research done at Felege Hiwot Compressive Hospital (47.7%) and a clinical trial conducted by Wang et al., respectively (Wang et al., 2015; Kefale et al., 2020). None of our patients received thrombolytic therapy because of its unavailability. In the multivariable analysis, the probability of an adverse outcome was twice as high among patients aged 45–64 years compared to those younger than 45 years of age. This finding was in line with studies conducted in India in 2015 and in Tikur Anbessa Specialized Hospital, Addis Ababa, Ethiopia (Krishnamurthi et al., 2015; Kassaw Asres et al., 2020). A possible explanation for this association might be due to their vulnerabilities and higher chances of risky exposures; practicing multiple risky behaviors, such as alcohol drinking, Khat chewing, and smoking cigarettes; and many other risky behaviors and being less likely to adhere to therapy that certainly increases the chance of having poor treatment outcomes.

Several studies have revealed that the presence of stroke-associated pneumonia, a low GCS score (<8), and high blood pressure at admission are significantly associated with poor treatment outcomes (Atadzhanov et al., 2012; Asgedom et al., 2020; Kassaw Asres et al., 2020), which is similar to our study findings. This might be due to poor health care delivery systems and facilities in sub-Saharan African countries, which are not capable of early diagnosis and proper treatment of such patients.

This study found that patients with poor treatment outcomes were three times more likely to have a history of renal injury (serum creatinine level > 1). This finding is supported by a study on acute kidney injury after stroke, which showed that acute kidney injure (AKI) after a stroke is linked with increased hospital death in 1 month after discharge and persistent disability (Huang et al., 2020).

## Conclusion

More than half of all stroke patients hospitalized had poor treatment outcomes. The most prevalent form of stroke in the hospitals is ischemic stroke. In addition, most patients were admitted to the hospital within 24 h of the onset of symptoms. Aspirin, statins, and antihypertensive medication were commonly ordered medications in stroke treatment. Moreover, age, uncontrolled hypertension, GCS score at admission, and serum creatine have been the most identified factors that have significant associations with poor treatment outcomes in stroke patients. Finally, we recommend that a community-based stroke prevention program should give priority to screening and management of hypertension and awareness creation about the benefits of treatment adherence is recommended, as this is the most prevalent comorbidity, as well as risk factors identified for poor treatment outcome in this study.

## Limitations of the study

The study's drawback is that it was done at a single hospital in eastern Ethiopia; therefore, the findings cannot be applied to all stroke patients in Ethiopia. Data from patients' medical records were inadequate to evaluate outcome variables objectively using outcome scores such as the Modified Ranking Scale; hence, the outcome of interest was measured subjectively.

## Data availability statement

The original contributions presented in the study are included in the article/supplementary material, further inquiries can be directed to the corresponding author.

## Ethics statement

The studies involving humans were approved by Ethical clearance was obtained from the Institutional Health Research Ethics Review Committee (IHRERC) of College of Health and Medical Sciences, Haramaya University. The studies were conducted in accordance with the local legislation and institutional requirements. The participants provided their written informed consent to participate in this study.

## Author contributions

ZA: Conceptualization, Data curation, Investigation, Methodology, Software, Writing—original draft, Writing—review & editing. SM: Supervision, Validation, Visualization, Writing—review & editing. AA: Supervision, Validation, Visualization, Writing—review & editing. TW: Supervision, Validation, Visualization, Writing—review & editing. AB: Data curation, Investigation, Software, Writing—review & editing. BM: Conceptualization, Data curation, Investigation, Methodology, Software, Writing—review & editing.

## References

[B1] Abate T. W. Zeleke B. Genanew A. Abate B. W. (2021). The burden of stroke and modifiable risk factors in Ethiopia: a systemic review and meta-analysis. PloS ONE 16, e0259244. 10.1371/journal.pone.0259244 34723996 PMC8559958

[B2] Abbasi M. Y. Ali M. A. (2012). Prescribing pattern of drugs in stroke patients: a prospective study. Arch. Pharm. Prac. 3, 1–4. 10.4103/2045-080X.106253

[B3] Akinyemi R. O. Ovbiagele B. Adeniji O. A. Sarfo F. S. Abd-Allah F. Adoukonou T.. (2021). Stroke in Africa: profile, progress, prospects and priorities. Nat. Rev. Neurol. 17, 634–656. 10.1038/s41582-021-00542-4 34526674 PMC8441961

[B4] Alene M. Assemie M. A. Yismaw L. Ketema D. B. (2020). Magnitude of risk factors and in-hospital mortality of stroke in Ethiopia: a systematic review and meta-analysis. BMC Neurol. 20, 1–10. 10.1186/s12883-020-01870-6 32814556 PMC7437163

[B5] Asgedom S. W. Gidey K. Gidey K. Niriayo Y. L. Desta D. M. Atey T. M.. (2020). Medical complications and mortality of hospitalized stroke patients. J. Stroke Cereb. Dis. 29, 104990. 10.1016/j.jstrokecerebrovasdis.2020.104990 32689635

[B6] Atadzhanov M. Mukomena N. P. Lakhi S. A. Ross O. F. Meschia J. (2012). Stroke characteristics and outcomes of adult patients admitted to the university teaching hospital, Lusaka, Zambia. The Open Gen. Int. Med. J. 5, 1–21. 10.2174/1874076601205010003

[B7] Avan A. Digaleh H. Di Napoli M. Stranges S. Behrouz R. Shojaeianbabaei G.. (2019). Socioeconomic status and stroke incidence, prevalence, mortality, and worldwide burden: an ecological analysis from the global burden of disease study 2017. BMC Med. 17, 191. 10.1186/s12916-019-1397-3 31647003 PMC6813111

[B8] Baye M. Hintze A. Gordon-Murer C. Mariscal T. Belay G. J. Gebremariam A. A.. (2020). Stroke characteristics and outcomes of adult patients in Northwest Ethiopia. Front. Neurol. 11, 428. 10.3389/fneur.2020.00428 32508740 PMC7248259

[B9] Bindslev J. B. Johnsen S. P. Hansen K. Valentin J. B. Hoei-Hansen C. E. Truelsen T.. (2023). The positive predictive value of pediatric stroke diagnoses in administrative data: a retrospective validation study. Clin. Epidemiol. 31, 755–764. 10.2147/CLEP.S414913 37360512 PMC10290464

[B10] De Wit L. Theuns P. Dejaeger E. Devos S. Gantenbein A. R. Kerckhofs E.. (2017). Long-term impact of stroke on patients' health-related quality of life. Disab. Rehab. 39, 1435–1440. 10.1080/09638288.2016.1200676 27385479

[B11] Deresse B. Shaweno D. (2015). Epidemiology and in-hospital outcome of stroke in South Ethiopia. J. Neurol. Sci. 355, 138–142. 10.1016/j.jns.2015.06.001 26059446

[B12] Fekadu G. Adola B. Mosisa G. Shibiru T. Chelkeba L. (2020). Clinical characteristics and treatment outcomes among stroke patients hospitalized to Nekemte referral hospital, western Ethiopia. J. Clin. Neurosci. 71, 170–176. 10.1016/j.jocn.2019.08.075 31471079

[B13] Gadisa D. A. Busawa G. B. Gebremariam E. T. Tefera G. M. Belete K. T. Taye G. M.. (2021). Clinical characteristics, treatment outcomes, and its predictors among hospitalized stroke patients in ambo university referral hospital, West Ethiopia: a retrospective hospital-based study. Vascular Health Risk Manage. 5, 591–604. 10.2147/VHRM.S287465 33447039 PMC7802015

[B14] Hilari K. (2011). The impact of stroke: are people with aphasia different to those without? Disab. Rehab. 33, 211–218. 10.3109/09638288.2010.508829 20712416

[B15] Huang Y. Wan C. Wu G. (2020). Acute kidney injury after a stroke: a PRISMA-compliant meta-analysis. Brain Behav. 10, e01722. 10.1002/brb3.1722 32757350 PMC7507391

[B16] Johnson W. Onuma O. Owolabi M. Sachdev S. (2016). Stroke: a global response is needed. Bull World Health Organ. 94, 634. 10.2471/BLT.16.181636 27708464 PMC5034645

[B17] Kassaw Asres A. Cherie A. Bedada T. Gebrekidan H. (2020). Frequency, nursing managements and stroke patients' outcomes among patients admitted to Tikur Anbessa specialized hospital, Addis Ababa, Ethiopia a retrospective, institution based cross-sectional study. Int. J. Africa Nurs. Sci. 13, 100228. 10.1016/j.ijans.2020.100228

[B18] Kefale B. Ewunetei A. Molla M. Tegegne G. T. Degu A. (2020). Clinical pattern and predictors of stroke treatment outcome among hospitalised patients who had a stroke at Felege Hiwot comprehensive specialised hospital, northwest Ethiopia: a retrospective cross-sectional study. BMJ Open 10, e040238. 10.1136/bmjopen-2020-040238 33384388 PMC7780509

[B19] Krishnamurthi R. V. Moran A. E. Feigin V. L. Barker-Collo S. Norrving B. Mensah G. A.. (2015). Stroke prevalence, mortality and disability-adjusted life years in adults aged 20-64 years in 1990-2013: data from the global burden of disease 2013 study. Neuroepidemiology 45, 190–202. 10.1159/000441098 26505983

[B20] Mairami F. F. Warren N. Allotey P. A. Mak J. S. Reidpath D. D. (2020). Documenting the impact of stroke in a middle-income country: a Malaysian case study. Disab. Rehab. 42, 102–113. 10.1080/09638288.2018.1493544 30183424

[B21] Mulugeta H. Yehuala A. Haile D. Mekonnen N. Dessie G. Kassa G. M.. (2020). Magnitude, risk factors and outcomes of stroke at Debre Markos Referral Hospital, Northwest Ethiopia: a retrospective observational study. The Egyptian J. Neurol. Psychiatry Neurosurg. 56, 1–9. 10.1186/s41983-020-00173-4

[B22] Owolabi M. O. Arulogun O. Melikam S. Adeoye A. M. Akarolo-Anthony S. Akinyemi R.. (2015). The burden of stroke in Africa: a glance at the present and a glimpse into the future. Cardiovasc. J. Afr. 26, S27–38. 10.5830/CVJA-2015-038 25962945 PMC4557491

[B23] Satink T. Cup E. H. Ilott I. Prins J. De Swart B. J. (2013). Patients' views on the impact of stroke on their roles and self: a thematic synthesis of qualitative studies. Arch. Phys. Med. Rehab. 94, 1171–1183. 10.1016/j.apmr.2013.01.011 23337428

[B24] Shenkutie Greffie E. (2015). Risk factors, clinical pattern and outcome of stroke in a referral hospital, northwest Ethiopia. CMR 4, 182. 10.11648/j.cmr.20150406.13

[B25] Tesfaye B. (2017). Assessment of Risk Factors and Treatment Outcome of Stroke Admissions at St. Paul's Teaching Hospital, Addis Ababa, Ethiopia. J. Neurol. Neurophysiol. 12, 8. 10.4172/2155-9562.1000431

[B26] van Eeden M. van Heugten C. M. Evers S. M. (2012). The economic impact of stroke in The Netherlands: the €-restore4stroke study. BMC Pub. Health 12, 1–2. 10.1186/1471-2458-12-122 22329910 PMC3305460

[B27] Wang X. Arima H. Yang J. Zhang S. Wu G. Woodward M.. (2015). Mannitol and outcome in intracerebral hemorrhage: propensity score and multivariable intensive blood pressure reduction in acute cerebral hemorrhage trial 2 results. Stroke 46, 2762–2767. 10.1161/STROKEAHA.115.009357 26265125

[B28] Zewudie A. Z. Regasa T. Hambisa S. Nureye D. Mamo Y. Aferu T.. (2020). Treatment outcome and its determinants among patients admitted to stroke unit of jimma university medical center, southwest Ethiopia. Stroke Res. Treatment 30, 2020. 10.1155/2020/8817948 33489080 PMC7790566

